# Hospital Meetings

**Published:** 1896-06-27

**Authors:** 


### HOSPITAL MEETINGS.

### METROPOLITAN HOSPITAL.

The Hon. Vicary Gibbs, M.P., presided at the festival dinner of the Metropolitan Hospital, which was held at the Whitehall Rooms on the 23rd inst. This hospital was founded in 1836, and was for many years situated in Devonshire-square, in the City of London. Owing to railway extensions and other causes the committee were compelled to seek another site, and finding that the north-eastern portion of London was almost destitute of hospital accommodation the present hospital was erected in Kingsland-road, with accommodation for one hundred and sixty patients. Only sixty-six of these beds, however, are open at the present time owing to lack of funds. At the dinner the Chairman mentioned that the hospital lacked one important thing—what Shakespeare called "those rascally counters." That was all it wanted to make it the most efficient and valuable hospital in the great City of London. It had a splendid building in an excellent site for its purposes, but it had never been able to open more than seventy-six beds, although it could for a comparatively small amount double its usefulness. He most earnestly urged the claims of this hospital, which, although admirably placed for its work, was not well placed for the purposes of advertisement. Other great metropolitan hospitals were well situated, but the Metropolitan Hospital was erected in a neighbourhood where there were very few rich people. In order to minister to the poor the hospital had removed its quarters from within the boundaries of the City, and it was to be hoped that this would not be allowed to react to its prejudice, for within its doors were treated many who worked in the City. The Chairman thought it was a good omen that so much money had been found for Guy's Hospital, for it showed that the springs of charity had not run dry. (Applause.)—Lord Battersea, in a humorous speech, said that the hospital wanted more than the subscriptions of the City, which were generous enough; it wanted the subscriptions of the people at large, and of the West-end in particular. He added that there was no hospital in London where the Jews could receive treatment such as they received at the Metropolitan Hospital. One of the chief merits of the hospital was its provident side. The hospital was worked on the system that it was essential that those who could pay something towards their treatment should do so—a system which he believed was at the root of the future of all the hospitals of London. This institution was the first which established that provident system, and the number who subscribed in the neighbourhood was increasing year by year. In fact, he hoped that the time was not far distant when the whole cost of the out-patient department, and a considerable proportion of the expenses of the in-patients would be met by the patients' payments.—£2,762 was subscribed as a result of the dinner.

### ST. MARY'S HOSPITAL.

The festival dinner in aid of the funds of the Clarence Memorial Wing of the St. Mary's Hospital was held at the Whitehall Rooms on the 17th inst., Sir William Broadbent, Bart., the senior physician of the hospital, in the chair. This extension, the building of which was commenced about 18 months ago, will comprise a new out-patients' department, a nurses' home, lying-in wards, various special wards, and a resident college for students and resident officers. The construction of the basement of the new building has exhausted the whole of the funds (some £14,C00) available for building, and the dinner formed part of the efforts of the managers to provide a further £12,500 to complete the first portion—the out-patients' department—the necessity for which is rendered more urgent every year by the ever-increasing number of patients. The Chairman, in the course of the evening, said that it was a popular notion that the training of medical men at a hospital was done at the expense of the patients. That was a mistaken idea. The welfare of the patients was the first consideration. The presence of students in the wards was really a definite advantage to the patients. It cheered them up, and brought them into closer relation with the outer world. Almost for the first time in their lives they found themselves the subjects of great interest—he had actually known serious illness cured by the simple fact of the examination the patients had undergone and the interest their case had excited. The students then, as he had before said, developed an air of cheerfulness in the wards which could only be appreciated by those who had gone round the gloomy wards of workhouse infirmaries. Besides this a medical school was actually a saving to a hospital, for the hospital obtained the services of competent men for no salary at all, and the patients were thus enabled to receive an amount of individual care and attention which would otherwise be absolutely impossible. The result of the dinner was very satisfactory, the subscription list amounting to about £4,800.

